# A Study on Serum Cortisol Levels and Thyroid Profile in Patients With Unipolar Depression

**DOI:** 10.7759/cureus.72125

**Published:** 2024-10-22

**Authors:** Gulfisha Aqeel, Atosh Kumar, Samarjeet Kaur, Munish Rastogi, Dolly Rastogi, Jayvardhan Singh

**Affiliations:** 1 Physiology, Ganesh Shankar Vidyarthi Memorial Medical College, Kanpur, IND; 2 Community Medicine, Ganesh Shankar Vidyarthi Memorial Medical College, Kanpur, IND; 3 Medical Microbiology, Institute of Health Sciences, Chhatrapati Shahu Ji Maharaj University, Kanpur, IND

**Keywords:** ham-d scale, serum cortisol, thyrotropin, thyroxine, triiodothyronine

## Abstract

Background

The hypothalamic-pituitary-adrenal (HPA) axis and the hypothalamic-pituitary-thyroid (HPT) axis are two neuroendocrine systems that play a crucial role in maintaining homeostasis and regulating mood alterations. These axes have also been implicated in the development and progression of unipolar depression (UD). This study aims to investigate the role of serum cortisol and thyroid function tests on the severity of depression among patients with UD attending the psychiatry department. The severity of depression was assessed using the Hamilton depression rating scale (HAM-D).

Methodology

In this cross-sectional study, 58 cases of UD were taken, and their serum cortisol levels and thyroid function tests were compared with the same-age and sex-matched controls with no psychiatric disorder.

Result

The serum cortisol levels were high among patients with UD as compared to controls, but these high values were not statistically significant (p-value >0.05). Serum triiodothyronine (T3) and thyroxine (T4) were lower among patients with UD as compared to controls. Serum thyrotropin (TSH) levels were significantly higher among patients with UD as compared to controls. Regression analysis revealed a linear relationship between levels of serum TSH and HAM-D score.

Conclusion

In conclusion, our study emphasizes the significance of the thyroid profile, especially TSH levels, in relation to depression and its severity.

## Introduction

Depressive disorder, also known as unipolar depression (UD) or major depressive disorder (MDD), is a severe mental health disorder characterized by continuous feelings of sadness, hopelessness, and loss of interest in activities [1]. The prevalence of UD in India is estimated between 3% and 16.9%, while the worldwide prevalence ranges from 3% to 10% [2,3].

The exact pathophysiology of UD remains unclear; recent evidence suggests that abnormalities in the hypothalamic-pituitary-adrenal (HPA) axis and hypothalamic-pituitary-thyroid (HPT) axis may play a crucial role in its development [4]. The HPA axis is a critical neuroendocrine system that regulates stress response, mood, and emotional behavior. Cortisol is the main hormone produced by the adrenal gland, which plays a central role in this axis. Elevated cortisol levels have always been observed in patients with UD, suggesting a state of hypercortisolemia. This hypercortisolemia may contribute to the development of depressive symptoms by disrupting neurotransmitter systems and neural circuits involved in mood regulation [5].

Thyroid hormones, particularly triiodothyronine (T3) and thyroxine (T4), also have an important role in mood regulation. Both hypothyroidism (underactive thyroid) and hyperthyroidism (overactive thyroid) can cause mood abnormalities, including depression. Hypothyroidism is distinguished by raised TSH levels and lowered T3 and T4 levels, while subclinical hypothyroidism is distinguished by raised TSH levels with normal T3 and T4 levels [6]. Altered thyroid-stimulating hormone (TSH) levels have been observed in patients with UD, suggesting potential disturbance in thyroid hormone regulation [7].

A complex interplay exists between the cortisol hormone and thyroid hormone regulation in UD thus, this study aims to explore the relationship between thyroid hormones (T3, T4, TSH) and cortisol levels to understand the gap that exists in the interaction between the HPT and HPA axis with UD. Additionally, it aims to investigate the association between serum cortisol and thyroid hormones with the severity of UD.

## Materials and methods

The present study was a cross-sectional, comparative study conducted at the Department of Physiology and Department of Psychiatry, GSVM Medical College, Kanpur, India, over one year, from July 2023 to June 2024. The sample size for the study was calculated with reference to a previously published study [8]. This study reported a 26.2% prevalence of thyroid disorder among patients with UD and 7% in the general population. Thus, the sample size of 58 for each group was calculated using open-source OpenEpi software (https://www.openepi.com/).

The inclusion criteria for the cases required participants to be between 18 and 60 years old and meet the diagnostic criteria for UD. The diagnosis was confirmed by a psychiatrist based on the Diagnostic and Statistical Manual of Mental Disorders (DSM-5). To further evaluate the severity of depression, the 17-item Hamilton Rating Scale for Depression (HAM-D), a widely used clinician-rated tool, was employed to assess the severity in patients diagnosed with depression. Participants were required to have no clinical manifestations of thyroid or adrenocortical disease and must not have been on any medications known to interfere with HPA or HPT axis, such as thyroid hormones, amiodarone, or corticosteroids. Apparently, healthy controls with similar age and sex profiles were selected without any history of psychiatric disorders to serve as a comparison group. Written informed consent was obtained from all participants before the commencement of the study.

Exclusion criteria for both cases and control were bipolar disorder or any other psychiatric illnesses, clinically manifest thyroid or adrenocortical disease, pregnancy, and those who received any corticosteroids, drugs interfering with thyroid hormone metabolism, thyroid hormone, psychotropic medications, or antidepressive therapies during the last three months before inclusion. Demographic details, including the age, sex, and medical history of each participant, were recorded on a predesigned, pretested questionnaire.

Ethical approval for this study was obtained from the Institutional Ethics Committee, GSVM, Medical College, Kanpur (EC/BMHR/2022/172, Dated 29-12-2022). Informed consent was obtained from each participant before their inclusion in the study. Confidentiality and data protection protocols were strictly adhered to throughout the research process.

Blood samples were collected from each participant in the morning to minimize diurnal variations in cortisol levels. Morning blood samples on fasting of about 10-12 hours were taken between 8:00 a.m. and 10:00 a.m. A venipuncture was used to draw 5 mL of venous blood from each participant under aseptic conditions to measure thyroid profile and serum cortisol. The blood sample collected was allowed to clot by placing it in a rack at room temperature for at least 30 minutes and a maximum of one hour. Then, it was centrifuged at 3,000 rpm for five minutes, and the separated serum sample was stored at -20 degrees Celsius until used. Proper labeling and documentation of the samples were maintained to ensure accurate tracking and analysis. The clear serum obtained from the whole blood was analyzed for TSH, free T3, free T4, and serum cortisol on cobas e 402 analytical unit (Roche Diagnostics, Basel, Switzerland) using electrochemiluminescence immunoassay (ECLIA) technology. All the assays were performed strictly following the manufacturer's instructions. Quality control measures were implemented throughout the study, including regular calibration of laboratory equipment, adherence to standard operating procedures, and the inclusion of internal controls to ensure the accuracy and reliability of the results.

Continuous variables were presented as mean ± standard deviation (SD) and were compared by Student’s independent t-test. Categorical data were indicated by numbers and percentages (%) and were further compared by the chi-square test. Multiple linear regression was conducted to examine the effect of predictors (serum cortisol, free T3, free T4, and TSH) on HAM-D; for each predictor, estimate, standard error (SE), t-value, p-value, and adjusted R square were calculated. A p-value of <0.05 was considered statistically significant. All analyses were performed using the open-source statistical software Jamovi (version 2.3.13.0; https://www.jamovi.org/).

## Results

The study included 58 newly diagnosed patients of UD as cases and 58 age, sex-matched healthy controls. The mean age (years) in the case group was 38 ± 7.89, while, in the control group, it was 39.6 ± 7.11. There was no significant difference between the age and sex of cases and controls.

The mean serum cortisol (ng/dL) value in cases was 11 ± 5.11, while, in controls, it was 9.97 ± 4.32 (p > 0.05); though it is comparatively high in cases, there is no statistically significant mean difference in serum cortisol values of cases and controls. Mean serum T3 (ng/mL) in cases and control are 132 ± 9.88 and 135 ± 9.78, respectively (p > 0.05). Mean values of serum T4 (ng/mL) in cases and controls are 7.92 ± 0.96 and 8.12 ± 0.86, respectively (p > 0.05). Mean values of serum TSH (µU/mL) in cases and controls were 4.72 ± 2.71 and 3.1 ± 0.9, respectively (p < 0.05). Thus, among serum cortisol, T3, T4, and TSH, only TSH shows a statistically significant difference in its value between cases and controls. The mean HAM-D score values among cases came out to be 16.2 ± 3.52, while its value in controls was 11.4 ± 1.94 (p < 0.05). Thus, it shows a statistically significant difference (Table 1).

**Table 1:** Sociodemographic, biochemical, and clinical factors of study participants The above table compares sociodemographic, biochemical, and clinical factors between cases and controls using Student's independent t-test. BMI: Body mass index; TSH: Thyroid-stimulating hormone; T3: Triiodothyronine; T4: Thyroxine; HAM-D score: Hamilton Depression Rating Scale

Study Variables	Cases (n=58)	Controls (n=58)	p-value
Age (years)	38 ± 7.89	39.6 ±7.11	>0.05
BMI (kg/m^2^)	22.6 ± 1.52	22.7 ± 1.47	>0.05
Serum Cortisol (ng/dL)	11 ± 5.11	9.97 ± 4.32	>0.05
T3 (ng/dL)	132 ± 9.88	135 ± 9.78	>0.05
T4 (ng/dL)	7.92 ± 0.96	8.12 ± 0.86	>0.05
TSH (µIU/mL)	4.72 ± 2.71	3.1 ± 0.9	<0.05
HAM-D score	16.2 ± 3.52	11.4 ± 1.94	<0.05

The outcome of multiple linear regression to examine the effect of predictors (serum cortisol, T3, T4, and TSH) on HAM-D indicates that TSH is a significant predictor of HAM-D scores, implying a potential link between increased TSH levels and severity of depression. However, other predictors, including cortisol, T3, and T4, did not show significant associations (Table 2).

**Table 2:** Regression analysis of HAM-D scores with hormonal predictors The above table shows the outcomes of multiple linear regression analysis between predictors (serum cortisol, T3, T4, TSH) and HAM-D score. TSH: Thyroid-stimulating hormone; T3: Triiodothyronine; T4: Thyroxine

Predictors	Estimate	Standard Error (SE)	t-value	P value
Serum cortisol	0.00	0.07	-0.05	>0.05
TSH	0.63	0.16	3.99	<0.001
T4	-0.29	0.38	-0.75	>0.05
T3	0.04	0.03	1.16	>0.05

**Figure 1:**
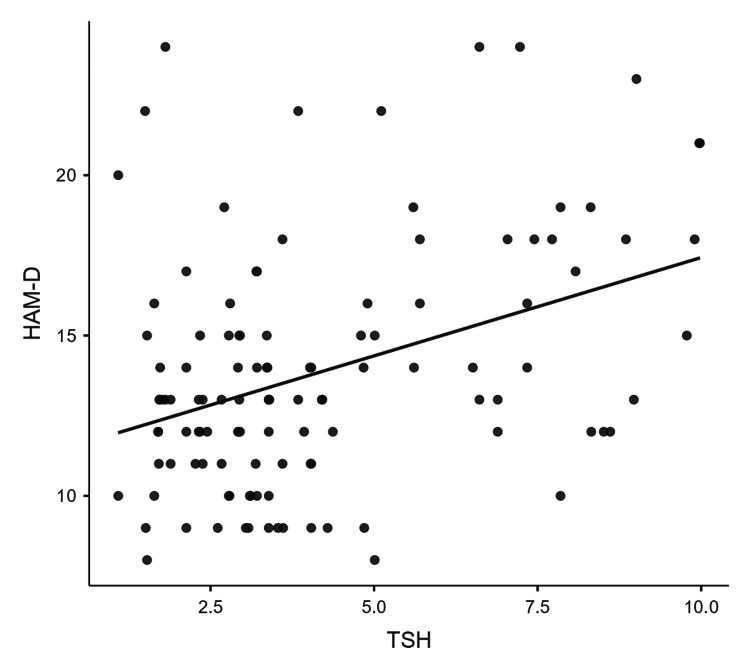
Scatter plot illustrating a positive correlation between TSH levels and HAM-D scores A straight regression line suggests a linear relationship between TSH levels and depression severity (HAM-D scores). TSH: Serum thyrotropin; HAM-D: Hamilton depression rating scale

Figure 1 illustrates a scatter plot showing a positive correlation between serum TSH and HAM-D scores, signifying an increase in the severity of depression with serum TSH.

## Discussion

This study aimed to explore the effect of serum cortisol, T3, T4, and TSH on UD and further investigate the relationship between the roles of these hormones on the severity of depression, which was assessed by the HAM-D score. In our study, we observed that serum TSH levels were statistically different in cases and controls, while there was no difference in serum cortisol, T3, and T4, though mean serum cortisol was slightly higher in cases as compared to controls. It was also observed that TSH is a significant predictor of severity, while cortisol, T3, and T4 have no effect on the severity of depression.

The finding that cortisol levels had no significant difference in cases and controls is supported by studies assessing the effect of cortisol on depression by Chan et al. and Doan et al. [9,10], who found that serum cortisol levels had no significant effect on depression. A systematic review and meta-analysis conducted by Sahu et al., to compare cortisol levels in patients with depression versus healthy controls, indicated statistically significant higher levels of serum cortisol among depressed patients. Though this study had a substantial number of participants (1,400), the high heterogeneity (I2 = 85%) suggested variability in the results across different studies [11]. Cortisol levels had no effect on the severity of depression, which aligns with the findings of a study done by Alenko et al. [7].

In the thyroid hormone profile, T3 and T4 were not significantly different in cases and controls, while TSH was significantly higher in cases compared to controls. Studies describing the same endocrinal alterations in patients of UD [3,12] support these findings. Our study indicates that higher levels of TSH are associated with higher HAM-D scores, emphasizing the fact that as the TSH level increases more severe the depression becomes. Similar findings are seen in studies done by Roa Dueñas et al. and Wu et al. [13,14].

Limitations

The study has some limitations; for example, its cross-sectional design makes it difficult to establish causality, and the small sample size (58 cases and 58 controls) limits the generalizability of the findings.

## Conclusions

In conclusion, our study emphasizes the significance of the thyroid profile, especially TSH levels, in relation to depression and its severity. Further research with a larger population and community-based design is needed to examine the changes in thyroid hormones and cortisol over time to establish the causal relationship between the HPA and HPT axes and depressive disorders. This will help in forming diagnostic markers for the severity of depression.
